# Methanol Extract of *Usnea barbata* Induces Cell Killing, Apoptosis, and DNA Damage against Oral Cancer Cells through Oxidative Stress

**DOI:** 10.3390/antiox9080694

**Published:** 2020-08-03

**Authors:** Jen-Yang Tang, Kuang-Han Wu, Yen-Yun Wang, Ammad Ahmad Farooqi, Hurng-Wern Huang, Shyng-Shiou F. Yuan, Ru-In Jian, Li-Yi Tsao, Po-An Chen, Fang-Rong Chang, Yuan-Bin Cheng, Hao-Chun Hu, Hsueh-Wei Chang

**Affiliations:** 1 Department of Radiation Oncology, Faculty of Medicine, College of Medicine, Kaohsiung Medical University, Kaohsiung 80708, Taiwan; reyata@kmu.edu.tw; 2 Department of Radiation Oncology, Kaohsiung Medical University Hospital, Kaohsiung 80708, Taiwan; 3 Graduate Institute of Medicine, College of Medicine, Kaohsiung Medical University, Kaohsiung 80708, Taiwan; u108500037@kmu.edu.tw; 4 School of Dentistry, College of Dental Medicine, Kaohsiung Medical University, Kaohsiung 80708, Taiwan; wyy@kmu.edu.tw; 5 Cancer Center, Kaohsiung Medical University Hospital, Kaohsiung 80708, Taiwan; yuanssf@kmu.edu.tw; 6 Center for Cancer Research, Kaohsiung Medical University, Kaohsiung 80708, Taiwan; 7 Department of Molecular Oncology, Institute of Biomedical and Genetic Engineering (IBGE), Islamabad 44000, Pakistan; farooqiammadahmad@gmail.com; 8 Institute of Biomedical Science, National Sun Yat-sen University, Kaohsiung 80424, Taiwan; sting@mail.nsysu.edu.tw; 9 Translational Research Center, Kaohsiung Medical University Hospital, Kaohsiung 80708, Taiwan; 10 Department of Biomedical Science and Environmental Biology, College of Life Science, Kaohsiung Medical University, Kaohsiung 80708, Taiwan; r07b46002@ntu.edu.tw (R.-I.J.); r08642006@ntu.edu.tw (L.-Y.T.); u105023038@kmu.edu.tw (P.-A.C.); 11 Graduate Institute of Natural Products, Kaohsiung Medical University, Kaohsiung 80708, Taiwan; aaronfrc@kmu.edu.tw (F.-R.C.); jmb@kmu.edu.tw (Y.-B.C.); 12 Institute of Medical Science and Technology, National Sun Yat-sen University, Kaohsiung 80424, Taiwan

**Keywords:** lichen, natural product, preferential killing, oxidative stress, apoptosis, DNA damage

## Abstract

Some lichens provide the resources of common traditional medicines and show anticancer effects. However, the anticancer effect of *Usnproliea barbata* (*U. barbata*) is rarely investigated, especially for oral cancer cells. The aim of this study was to investigate the cell killing function of methanol extracts of *U. barbata* (MEUB) against oral cancer cells. MEUB shows preferential killing against a number of oral cancer cell lines (Ca9-22, OECM-1, CAL 27, HSC3, and SCC9) but rarely affects normal oral cell lines (HGF-1). Ca9-22 and OECM-1 cells display the highest sensitivity to MEUB and were chosen for concentration effect and time course experiments to address its cytotoxic mechanisms. MEUB induces apoptosis of oral cancer cells in terms of the findings from flow cytometric assays and Western blotting, such as subG1 accumulation, annexin V detection, and pancaspase activation as well as poly (ADP-ribose) polymerase (PARP) cleavage. MEUB induces oxidative stress and DNA damage of oral cancer cells following flow cytometric assays, such as reactive oxygen species (ROS)/mitochondrial superoxide (MitoSOX) production, mitochondrial membrane potential (MMP) depletion as well as overexpression of γH2AX and 8-oxo-2′deoxyguanosine (8-oxodG). All MEUB-induced changes in oral cancer cells were triggered by oxidative stress which was validated by pretreatment with antioxidant *N*-acetylcysteine (NAC). In conclusion, MEUB causes preferential killing of oral cancer cells and is associated with oxidative stress, apoptosis, and DNA damage.

## 1. Introduction

Oral cancer is one of the common cancers worldwide. Chemotherapy is one of the strategies for oral cancer treatment, in addition to radiotherapy. Side effects on normal tissues or cells are frequently associated with chemotherapy [1] and limit its application for cancer therapy. This warrants drug discovery with little side effects.

Natural products contain a number of bioactive compounds that may act on diverse targets or display different mechanisms [2], leading to synergistic actions with anticancer function but little side effects on normal cells. For example, several natural products such as grape seed extracts [3], ethyl acetate extract of *Nepenthes adrianii* × *clipeata* [4], water extract of *Scutellaria baicalensis* [5], *Zelkova serrata* twig extract [6] were reported to provide cell killing effects against oral cancer cells with low cytotoxicity to normal cells. This shows that natural products have the potential for preferential killing in anticancer treatments.

At least 52 genera of lichens make up the resources of common traditional medicines worldwide [7]. Extracts of at least 75 lichens such as *Cladonia ragiferina*, *Cladonia sylvatica*, *Evernia prunastri, Usnea barbata* (*U. barbata*) [8], and others [9] show high antioxidant capacity and total phenolic content. Antioxidants are reported to have dual functions that are dose dependent, i.e., it shows healthy or harmful effects at normal or high doses [10], respectively. At high doses, the exogenous antioxidants may induce an overproduction of oxidative stress [10], e.g., causing cancer cells to die. For this anticancer potential, lichen extracts warrant detailed investigation.

Different geographic regions favor different lichen genera in traditional medicines but *Usnea* is the most common genus in use [7]. Several studies of *Usnea* extracts have reported anticancer effects. For example, methanol extract of *U. intermedia* Ach. was reported to induce cell killing of human breast and lung cancer cells [11]. Water extract of *U. longissima* Ach. also exhibits cell killing against human glioblastoma U87MG cells [12]. Methanol extract of *U. filipendula* Stirt can induce cell killing of human lung and liver cancer cells [13]. However, the anticancer effect of *U. barbata* is rarely investigated, especially for oral cancer cells.

The subject of this study is to evaluate the cell killing effect and explore its mechanism in oral cancer cells by using methanol extract of *U. barbata* (MEUB). Cell survival, flow cytometry, and Western blotting are applied to investigate the involvement of oxidative stress, apoptosis, and DNA damage in MEUB-treated oral cancer cells.

## 2. Materials and Methods

### 2.1. Research Materials

Specimens of *U. barbata* were identified by Mr. Hao-Chun Hu and purchased from a traditional herbal medicine pharmacy, Jakarta, Indonesia in July, 2014. A voucher specimen (code no. KMU-I-006) was deposited in the Graduate Institute of Natural Products, College of Pharmacy, Kaohsiung Medical University, Kaohsiung, Taiwan.

### 2.2. Extraction and Partition of Methanol Extract of U. barbata (MEUB)

The air-dried lichen, *U. barbata*, was extracted thrice by methanol at room temperature. The crude extract was partitioned between equal volume ethyl acetate and water to obtain organic and water layers. After evaporation, the organic layer was further partitioned between hexanes/methanol/water (4:3:1) to obtain the hexane layer and 75% MeOH layer (MEUB). This methanol extract of *U. barbata*, named here MEUB, was dissolved in dimethyl sulfoxide (DMSO) for drug treatments. All drug treatments contained the same concentration of DMSO (0.1%).

### 2.3. HPLC Analysis of MEUB

The Shimadzu high performance liquid chromatography (HPLC) system consisted of a LC-20AT solvent delivery unit, a SPD-M10A diode array detector, and a CBM-20A controller. A C_18_ column (250 × 4.6 mm, 5 μm, 1.0 mL/min, Cosmosil, Nacalai Tesque, Kyoto, Japan) was selected for separation. The chromatography methods were listed below: Solution A: 1% phosphoric acid_(aq)_; solution B: MeOH; flow rate: 0.7 mL/min; 0 min: 30% solution B, 0–14 min: 30% to 70% solution B, 14–44 min: 70–100% solution B, 44–45 min: 100–30% solution B, 45–50 min: 30% solution B.

Usnic acid was regarded as the major component of supercritical CO_2_ extraction extracts of Lichen *U. barbara* [14]. Usnic acid also mainly occupies the dried weight of *U. florida* extract about 40% [15]. Accordingly, usnic acid (Sigma-Aldrich, St. Louis, MO, USA) was chosen as the reference of the putative main bioactive component in MEUB.

### 2.4. Determination of the Usnic Acid Content in MEUB

In the below HPLC chromatogram, nine different concentrations of usnic acid in linear range (0.001−0.01 mg/mL) were prepared in MeOH, respectively. Three replicates (*n* = 3) of each concentration were subjected to HPLC. The methods of the experimental section were performed according to our previous study [16].

### 2.5. Cell Culture and Free Radical Scavenger

Human oral cancer (Ca9-22, HSC3, CAL 27, and SCC9) and normal oral (HGF-1) cell lines were obtained from the Cell Bank, RIKEN BioResource Research Center (Tsukuba, Ibaraki, Japan) and American Type Culture Collection (ATCC, Manassas, VA, USA). Oral cancer OECM-1 cell lines [17] were a gift from Dr. Wan-Chi Tsai at Kaohsuing Medical University. All cells were humidifiedly incubated (37 °C, 5% CO_2_) as previously described [4]. *N*-acetylcysteine (NAC) (Sigma-Aldrich; St. Louis, MO, USA) was used as a free radical scavenger to examine the involvement of oxidative stress in different experiments [18,19,20].

### 2.6. Cell Viability

Cells were treated with MEUB (0 to 6 μg/mL) or cisplatin (0 to 4.5 μg/mL) for 48 h. For NAC group, cells were pre-treated with NAC (0 and 2 mM, 1 h) and post-treated with MEUB (48 h, 0 and 2 μg/mL or 0 to 6 μg/mL). Cell viability was analyzed by the tetrazolium dye MTS (Promega Corporation, Madison, WI, USA) as previously described [21].

### 2.7. Cell Cycle Assay

Cells were treated with MEUB (0 to 48 h, 2 μg/mL or 48 h, 0 to 6 μg/mL). For NAC group, cells were pre-treated with NAC (2 mM, 1 h) and post-treated with MEUB (48 h, 0 to 6 μg/mL or 0 to 48 h, 6 μg/mL). After drug treatments, cells were fixed with 75% ethanol. Cells were supplied with 7-aminoactinmycin D (7AAD) (Biotium, Inc., Hayward, CA, USA) [22] under the condition (1 μg/mL, 30 min) before cell cycle phase detection using Accuri C6 flow cytometry (Becton-Dickinson, Mansfield, MA, USA).

### 2.8. Annexin V/7AAD Apoptosis Assay

Cells were treated with MEUB (0 to 48 h, 6 μg/mL). For NAC group, cells were pre-treated with NAC (2 mM, 1 h) and post-treated with MEUB (48 h, 0 to 6 μg/mL). After drug treatments, cells were supplied with annexin V/7AAD reagents [23] (Strong Biotech Corporation, Taipei, Taiwan) following the manufacturer’s instructions before apoptosis detection using Accuri C6 flow cytometry.

### 2.9. Pancaspase Apoptosis Assay

Cells were treated with MEUB (0 to 48 h, 2 μg/mL or 48 h, 0 to 6 μg/mL). For NAC group, cells were pre-treated with NAC (2 mM, 1 h) and post-treated with MEUB (48 h, 0 to 6 μg/mL or 0 to 48 h, 2 μg/mL). After drug treatments, cells were supplied with the generic caspase activity reagents [21] (Abcam, Cambridge, UK) following the manufacturer’s instructions before apoptosis detection using Accuri C6 flow cytometry. This assay can measure caspases-1 and 3 to 9 activities based on the pan-reaction to their common peptide Val-Ala-Asp substrate [24].

### 2.10. Western Blotting for Apoptosis Signaling

Cells were treated with MEUB (48 h, 0 to 6 μg/mL or 0 to 48 h, 2 μg/mL). Detailed information of Western blotting was previously mentioned [25]. The primary apoptosis antibody against the cleaved form of poly (ADP-ribose) polymerase (c-PARP) (Cell Signaling Technology, Inc., Danvers, MA, USA) and internal control antibody against β-actin (Sigma-Aldrich, St. Louis, MO, USA) were applied.

### 2.11. ROS Assay

Cells were treated with MEUB (48 h, 0 to 6 μg/mL). For NAC group, cells were pre-treated with NAC (2 mM, 1 h) and post-treated with MEUB (0 to 6 h, 6 μg/mL). After drug treatments, cells were supplied with 2′,7′-dichlorodihydrofluorescein diacetate (DCFH-DA; Sigma-Aldrich) [26] under the condition (10 μM, 37 °C, 30 min) before ROS detection using Accuri C6 flow cytometry.

### 2.12. Mitochondrial Superoxide (MitoSOX) Assay

Cells were treated with MEUB (0 to 48 h, 6 μg/mL). For NAC group, cells were pre-treated with NAC (2 mM, 1 h) and post-treated with MEUB (48 h, 0 to 6 μg/mL). After drug treatments, cells were supplied with MitoSOX™ Red [27] (Molecular Probes, Invitrogen; Eugene, OR, USA) under the condition (50 nM, 30 min, 37°C) before MitoSOX detection using Accuri C6 flow cytometry.

### 2.13. Mitochondrial Membrane Potential (MMP) Assay

Cells were treated with MEUB (0 to 48 h, 6 μg/mL). For NAC group, cells were pre-treated with NAC (2 mM, 1 h) and post-treated with MEUB (48 h, 0 to 6 μg/mL). After drug treatments, cells were supplied with DiOC_2_(3) [28] (Invitrogen; San Diego, CA, USA) under the condition (5 nM, 30 min) before MMP detection using Accuri C6 flow cytometry.

### 2.14. γH2AX DNA Damage Assay

Cells were treated with MEUB (0 to 48 h, 2 μg/mL). For NAC group, cells were pre-treated with NAC (2 mM, 1 h) and post-treated with MEUB (48 h, 0 to 6 μg/mL). After drug treatments, cells were fixed with 75% ethanol. The primary antibody against γH2AX [29] (Santa Cruz Biotechnology; Santa Cruz, CA, USA) was incubated with fixed cells for 1 h at 4 °C. Alexa Fluor^®^488-conjugated secondary antibody (Cell Signaling Technology) was incubated for 30 min and 7AAD (1 μg/mL, 30 min) was applied before γH2AX detection using Accuri C6 flow cytometry.

### 2.15. 8-oxo-2′deoxyguanosine (8-oxodG) DNA Damage Assay

Cells were treated with MEUB (0 to 48 h, 2 μg/mL). For NAC group, cells were pre-treated with NAC (2 mM, 1 h) and post-treated with MEUB (48 h, 0 to 6 μg/mL). After drug treatments, cells were fixed with 75% ethanol. Cells were supplied with OxyDNA dye (EMD Millipore, Darmstadt, Germany) [23] under the condition (10× dilution, 1 h) before 8-oxodG detection using Accuri C6 flow cytometry.

### 2.16. Statistics

For multi-comparison, the data were analyzed by one-way analysis of variance (ANOVA) and Tukey HSD post hoc using JMP12 software (SAS Institute, Cary, NC, USA). Groups with different small letter labels differ significantly.

## 3. Results

### 3.1. HPLC Analysis of MEUB

**Figure 1:**
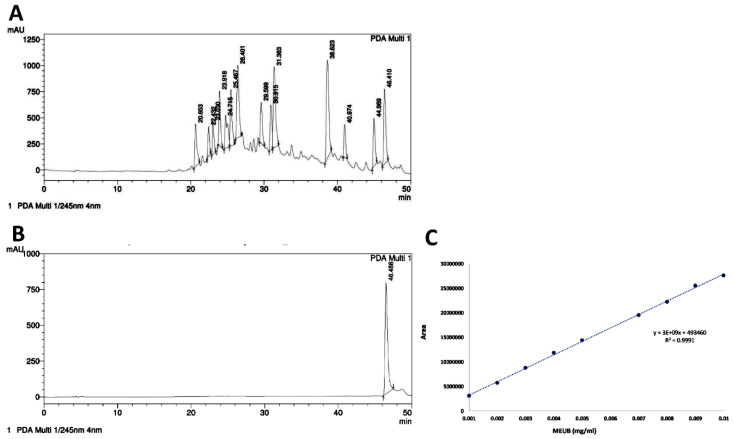
Fingerprint profile. (**A**) The HPLC-PDA fingerprint profile of *Usnproliea barbata* (MEUB). (**B**) The HPLC profile of usnic acid. (**C**) The calibration curve of usnic acid (*n* = 3).

Figure 1A shows the HPLC- Photo Diode Array (PDA) fingerprint profile of MEUB. Figure 1B shows the HPLC profile of usnic acid. The retention time for usnic acid is 46.488 min. The fingerprint profile of MEUB also shows the peak of usnic acid which is identified by NMR. During the calibration curve of usnic acid, the MEUB contains 3.16% usnic acid, i.e., 0.0316 mg usnic acid/mg MEUB (Figure 1C).

### 3.2. MEUB Sensitively Kills Oral Cancer Cells rather than Normal Oral Cells

**Figure 2:**
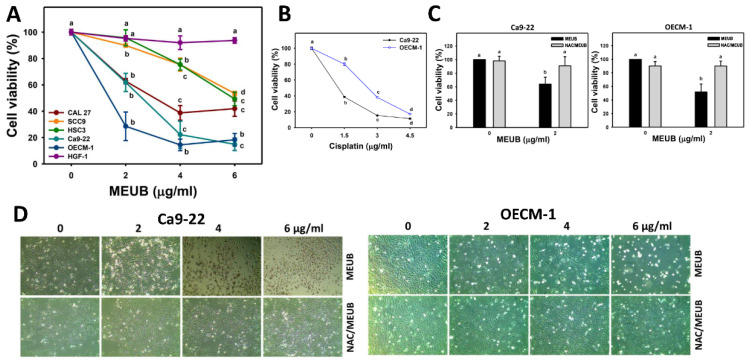
Cell viabilities and morphology of MEUB treatments in oral cancer cells. (**A**) Cell viability change of MEUB treatments. Oral cancer cells (CAL 27, SCC9, HSC3, Ca9-22, and OECM-1) and oral normal cells (HGF-1) were treated with MEUB (48 h, 0 to 6 μg/mL). MEUB (0 μg/mL) is the negative control containing 0.1% dimethyl sulfoxide (DMSO) in medium. (**B**) Cell viability change of cisplatin (48 h, 0 to 4.5 μg/mL) treatments. (**C**,**D**) Protective effects of *N*-acetylcysteine (NAC) pretreatment on cell viability and morphology in MEUB-treated oral cancer cells. Oral cancer cells were pre-treated with NAC (2 mM, 1 h) and post-treated with MEUB (0 and 2 μg/mL or 0 to 6 μg/mL) for 48 h. Data, mean ± SD (*n* = 3). Treatments labeled with different lower-case letters (a to d) indicate significant differences for multi-comparison. *p* < 0.05–0.0001.

As shown in Figure 2A, MEUB differentially decreases the MTS-detected cell viability (%) of five types of oral cancer cell lines, i.e., Ca9-22, OECM-1, CAL 27, HSC3, and SCC9. A cytotoxic side effect to normal cells was excluded by the finding that normal oral HGF-1 cells remain healthy to MEUB treatment within an amount of 6 μg/mL using MTS assay. Therefore, MEUB exhibits a preferential killing to oral cancer cells. Compared to other oral cancer cells, Ca9-22 and OECM-1 cells showed high sensitivity to MEUB. Both of them were selected for the following experiments to investigate the possible mechanism of preferential killing. For comparison, a clinical drug cisplatin was used as a positive control for oral cancer cell treatment (Figure 2B).

To validate the function of oxidative stress on the cell killing effect caused by MEUB, NAC pretreatment was used. In Figure 2C,D, MEUB-induced cell killing and abnormal morphology are inhibited by NAC in both Ca9-22 and OECM-1 cells.

### 3.3. MEUB Shows SubG1 Changes in Oral Cancer Cells

**Figure 3:**
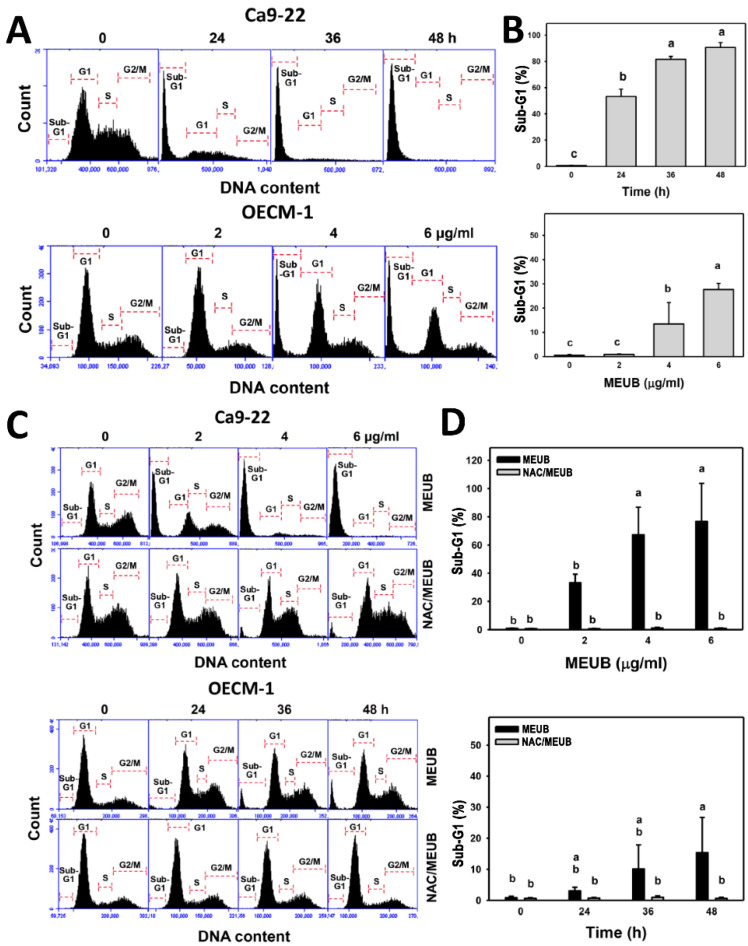
Cell cycle status of MEUB treatments in oral cancer cells. (**A**,**B**) Cell cycle patterns and statistics of MEUB treatments in oral cancer cells. Oral cancer cells were treated with MEUB, i.e., Ca9-22 cells (0 to 48 h, 2 μg/mL); OECM-1 cells (48 h, 0 to 6 μg/mL). MEUB (0 μg/mL) is the negative control containing 0.1% DMSO in medium. (**C**,**D**) Changes and statistics of NAC pretreatment on cell cycle distribution in MEUB-treated oral cancer cells. Oral cancer cells were pre-treated with NAC (2 mM, 1 h) and post-treated with MEUB, i.e., Ca9-22 cells (48 h, 0 to 6 μg/mL); OECM-1 cells (0 to 48 h, 6 μg/mL). Data, mean ± SD (*n* = 3). Treatments labeled with different lower-case letters (a–c) indicate significant difference for multi-comparison. *p* < 0.05–0.0001.

In Figure 3A, the cell cycle patterns in oral cancer cells after MEUB treatment are shown. In Figure 3B, different treatment times and concentrations of MEUB induce higher subG1 populations than control in oral cancer Ca9-22 and OECM-1 cells.

In Figure 3C, the cell cycle patterns in oral cancer cells after NAC pre-treatment and/or MEUB post-treatment are shown. In Figure 3D, different concentrations and treatment times of MEUB induce higher subG1 populations than control in oral cancer Ca9-22 and OECM-1 cells. Moreover, such MEUB-induced subG1 accumulation is suppressed by NAC pretreatment.

### 3.4. MEUB Shows Annexin V/7AAD-Detected Apoptosis in Oral Cancer Cells

**Figure 4:**
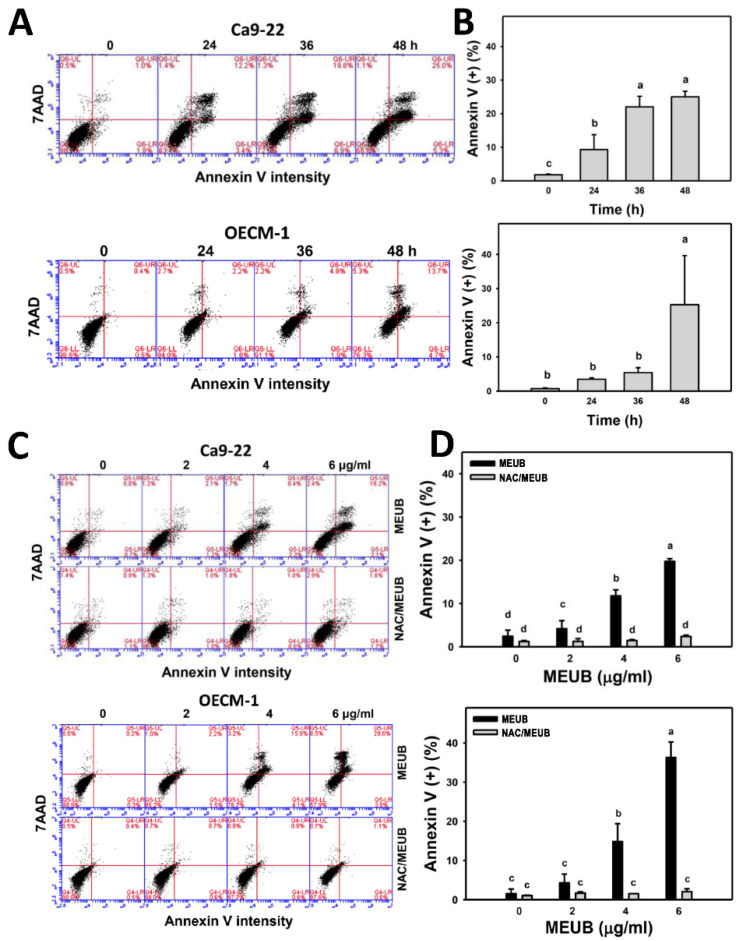
Apoptosis annexin V status of MEUB treatments in oral cancer cells. (**A**,**B**) Annexin V/7AAD pattern and statistics of MEUB treatments. Oral cancer cells (Ca9-22 and OECM-1) were treated with MEUB (0 to 48 h, 6 μg/mL). MEUB (0 μg/mL) is the negative control containing 0.1% DMSO in the medium. Annexin V (+)/7AAD (+ and −) was counted as apoptosis (+). (**C**,**D**) Protective effects and statistics of NAC pretreatment on apoptosis annexin V status in MEUB-treated oral cancer cells. Oral cancer cells were pre-treated with NAC (2 mM, 1 h) and post-treated with MEUB (48 h, 0 to 6 μg/mL). Data, mean ± SD (*n* = 3). Treatments labeled with different lower-case letters (a to d) indicate significant differences in this multi-comparison. *p* < 0.01–0.0001.

The fate of apoptosis in subG1 accumulation in oral cancer cells after MEUB treatment needed further confirmation by the annexin V/7AAD method. Here, the results are shown in Figure 4A. Different treatment times of MEUB induce higher annexin V-positive increment (apoptosis (+) (%)) than control in oral cancer Ca9-22 and OECM-1 cells (Figure 4B). Figure 4C shows the annexin V/7AAD patterns in oral cancer cells after NAC pre-treatment and/or MEUB post-treatment. In Figure 4D, different concentrations of MEUB induce higher apoptosis (+) (%) than control in oral cancer Ca9-22 and OECM-1 cells. Moreover, this MEUB-induced apoptosis (+) (%) is suppressed by NAC pretreatment.

### 3.5. MEUB Shows Pancaspase-Detected Apoptosis in Oral Cancer Cells

**Figure 5:**
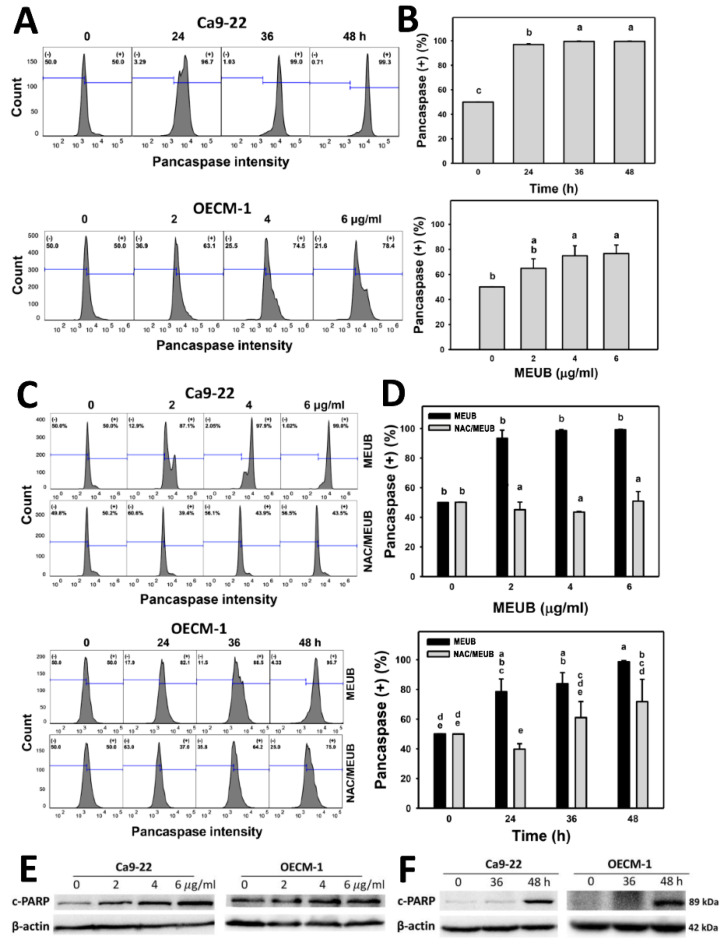
Caspase status of MEUB treatments in oral cancer cells. (**A**,**B**) Annexin V/7AAD pattern of MEUB treatments. Oral cancer cells (Ca9-22 and OECM-1) were treated with MEUB, i.e., Ca9-22 cells (0 to 48 h, 2 μg/mL); OECM-1 cells (48 h, 0 to 6 μg/mL). MEUB (0 μg/mL) is the negative control containing 0.1% DMSO in medium. Cells with high pancaspase intensity are marked by (+). (**C**,**D**) Protective effects and statistics of NAC pretreatment on the caspase status in MEUB-treated oral cancer cells. Oral cancer cells were pre-treated with NAC (2 mM, 1 h) and post-treated with MEUB, i.e., Ca9-22 cells (48 h, 0 to 6 μg/mL); OECM-1 cells (0 to 48 h, 2 μg/mL). Data are provided as mean ± SD (*n* = 3). Treatments labeled with different lower-case letters (a–e) indicate significant differences for multi-comparison. *p* < 0.05–0.0001. (**E**,**F**) Dose responses and time courses of MEUB on apoptosis protein expression in oral cancer cells. Oral cancer cells (Ca9-22 and OECM-1) were treated with MEUB (48 h, 0 to 6 μg/mL or 0 to 48 h, 2 μg/mL).

The annexin V apoptosis in oral cancer cells after MEUB treatment needed further confirmation by the pancaspase assay. In Figure 5A, pancaspase patterns in oral cancer cells are shown after MEUB treatment. In Figure 5B, different treatment times and concentrations of MEUB induce higher pancaspase activation (pancaspase (+) (%)) than control in oral cancer Ca9-22 and OECM-1 cells.

Figure 5C shows the pancaspase patterns in oral cancer cells after NAC pre-treatment and/or MEUB post-treatment. In Figure 5D, different concentrations and treatment times of MEUB induce higher pancaspase (+) (%) than control in oral cancer Ca9-22 and OECM-1 cells. Moreover, this MEUB-induced pancaspase (+) (%) is suppressed by NAC pretreatment.

### 3.6. MEUB Activates Apoptosis Signaling in Oral Cancer Cells

The annexin V apoptosis in oral cancer cells after MEUB treatment needed further confirmation by Western blotting-detecting apoptosis protein expressions. In Figure 5E, the different concentrations of MEUB induced an overexpression of cleaved PARP in oral cancer Ca9-22 and OECM-1 cells compared to the control. In Figure 5F, the MEUB-induced overexpression of cleaved PARP in oral cancer Ca9-22 and OECM-1 cells are compared to the control at different time intervals.

### 3.7. MEUB Shows ROS Induction in Oral Cancer Cells

**Figure 6:**
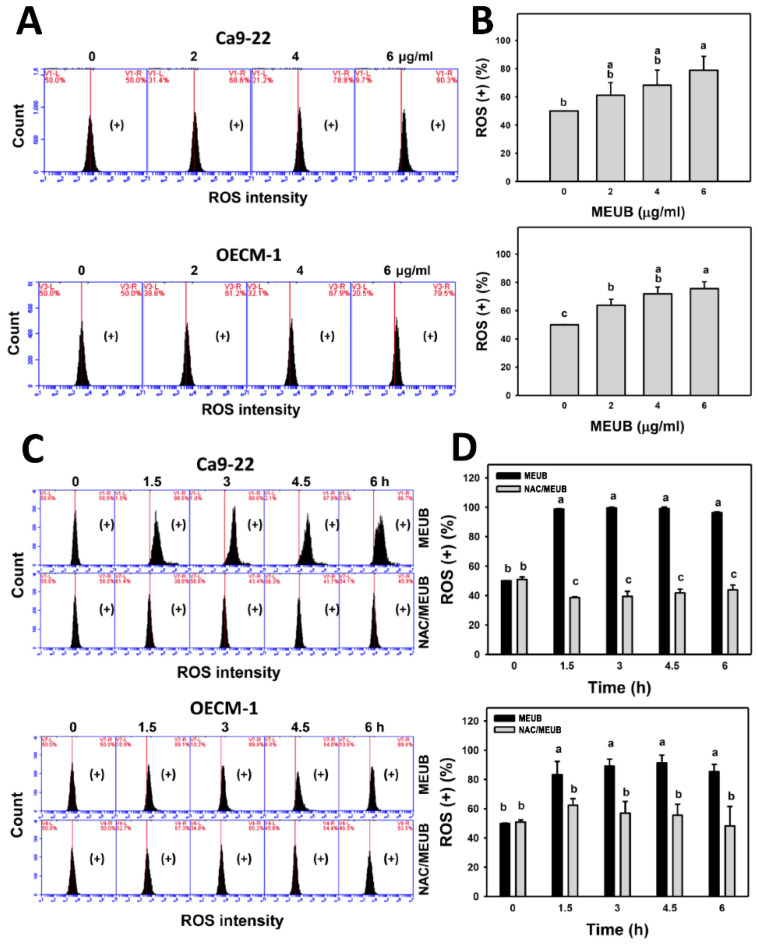
Reactive oxygen species (ROS) status of MEUB treatments in oral cancer cells. (**A**,**B**) ROS patterns of MEUB treatments. Oral cancer cells (Ca9-22 and OECM-1) were treated with MEUB (48 h, 0 to 6 μg/mL). MEUB (0 μg/mL) is the negative control containing 0.1% DMSO in the medium. Cells with high ROS intensity is marked in (+). (**C**,**D**) Protective effects and statistics of NAC pretreatment on ROS status in MEUB-treated oral cancer cells. Oral cancer cells were pre-treated with NAC (2 mM, 1 h) and post-treated with MEUB (0 to 6 h, 6 μg/mL). Data, mean ± SD (*n* = 3). Treatments labeled with different lower-case letters (a–c) indicate a significant difference in this multi-comparison. *p* < 0.05–0.0001.

The oxidative stress mechanism for cell killing effects of MEUB was further investigated by ROS measurement. In Figure 6A, ROS patterns in oral cancer cells after MEUB treatment are shown. In Figure 6B, different concentrations of MEUB induce higher ROS (+) (%) than control in oral cancer Ca9-22 and OECM-1 cells.

In Figure 6C, the ROS patterns in oral cancer cells are shown after NAC pre-treatment and/or MEUB post-treatment. In Figure 6D, different treatment times of MEUB induce higher ROS (+) (%) than control in oral cancer Ca9-22 and OECM-1 cells. Moreover, this MEUB-induced ROS (+) (%) is suppressed by NAC pretreatment.

### 3.8. MEUB Shows Superoxide Induction in Oral Cancer Cells

**Figure 7:**
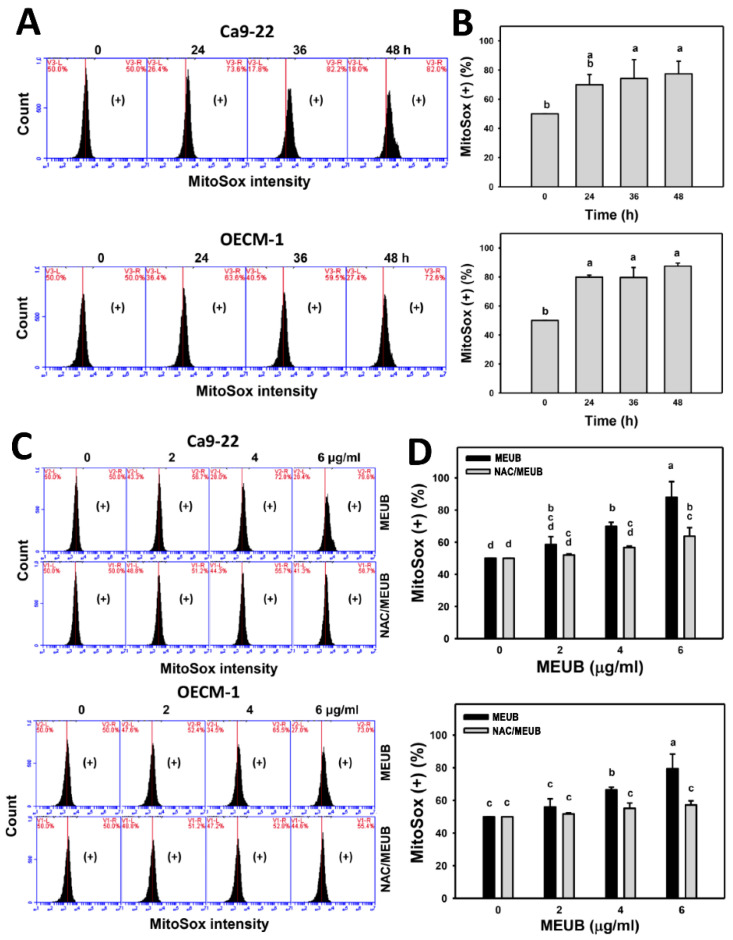
Mitochondrial superoxide (MitoSOX) status of MEUB treatments in oral cancer cells. (**A**,**B**) MitoSOX patterns of MEUB treatments. Oral cancer cells (Ca9-22 and OECM-1) were treated with MEUB (0 to 48 h, 6 μg/mL). MEUB (0 μg/mL) is the negative control containing 0.1% DMSO in the medium. Cells with high MitoSOX intensity is marked as (+). (**C**,**D**) Protective effects and statistics of NAC pretreatment on MitoSOX status in MEUB-treated oral cancer cells. Oral cancer cells were pre-treated with NAC (2 mM, 1 h) and post-treated with MEUB (48 h, 0 to 6 μg/mL). Data, mean ± SD (*n* = 3). Treatments labeled with different lower-case letters (a to d) indicate significant differences in this multi-comparison. *p* < 0.05–0.0001.

The oxidative stress mechanism for cell killing effects of MEUB was further investigated by mitochondrial superoxide (MitoSOX) measurement. In Figure 7A, MitoSOX patterns in oral cancer cells are shown after MEUB treatment. As shown in Figure 7B, different treatment times of MEUB induce higher MitoSOX (+) (%) than control in oral cancer Ca9-22 and OECM-1 cells.

Figure 7C shows the MitoSOX patterns in oral cancer cells after NAC pre-treatment and/or MEUB post-treatment. As shown in Figure 7D, different concentrations of MEUB induce higher MitoSOX (+) (%) than control in oral cancer Ca9-22 and OECM-1 cells. Moreover, the MEUB-induced MitoSOX (+) (%) is suppressed by NAC pretreatment.

### 3.9. MEUB Shows MMP Reduction in Oral Cancer Cells

**Figure 8:**
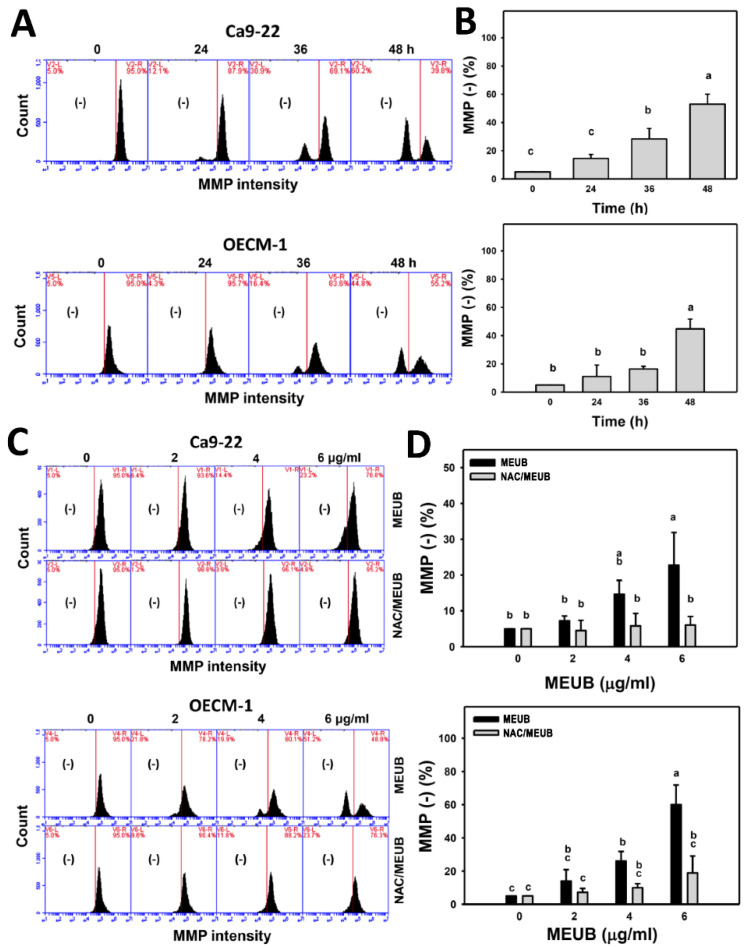
Mitochondrial membrane potential (MMP) status of MEUB treatments in oral cancer cells. (**A**,**B**) MMP patterns of MEUB treatments. Oral cancer cells (Ca9-22 and OECM-1) were treated with MEUB (0 to 48 h, 6 μg/mL). MEUB (0 μg/mL) is the negative control containing 0.1% DMSO in the medium. Cells with low MMP intensity are marked by (−). (**C**,**D**) Protective effects and statistics of NAC pretreatment on MMP status in MEUB-treated oral cancer cells. Oral cancer cells were pre-treated with NAC (2 mM, 1 h) and post-treated with MEUB (48 h, 0 to 6 μg/mL). Data, mean ± SD (*n* = 3). Treatments labeled with different lower-case letters (a–c) indicate significant differences in this multi-comparison. *p* < 0.05–0.0001.

The oxidative stress mechanism for cell killing effects of MEUB was further investigated by MMP measurement. Figure 8A shows the MMP patterns of oral cancer cells after MEUB treatment. Figure 8B shows that different treatment times of MEUB induce higher MMP (−) (%) than control in oral cancer Ca9-22 and OECM-1 cells.

In Figure 8C, the MMP patterns in oral cancer cells after NAC pre-treatment and/or MEUB post-treatment are shown. In Figure 8D, different concentrations of MEUB induce higher induce MMP (−) (%) than control in oral cancer Ca9-22 and OECM-1 cells. Moreover, this MEUB-induced MMP (−) (%) is suppressed by NAC pretreatment.

### 3.10. MEUB Shows γH2AX DNA Damage Induction in Oral Cancer Cells

**Figure 9:**
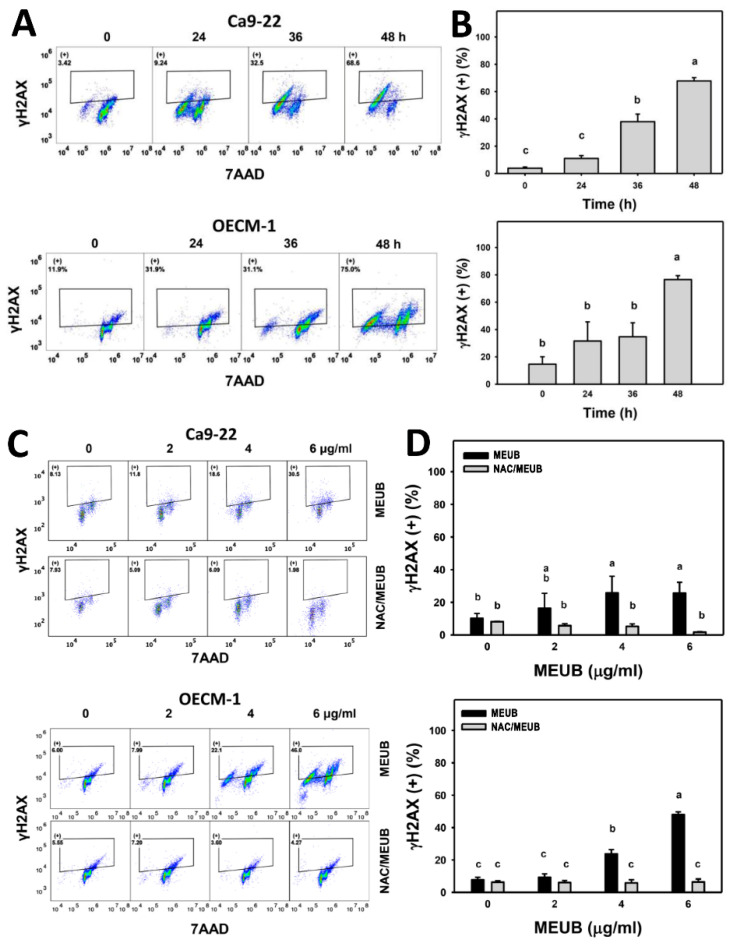
DNA double strand break status of oral cancer cells in MEUB treatments. (**A**,**B**) γH2AX patterns of MEUB treatments. Oral cancer cells (Ca9-22 and OECM-1) were treated with MEUB (0 to 48 h, 2 μg/mL). MEUB (0 μg/mL) is the negative control containing 0.1% DMSO in the medium. Cells with high γH2AX intensity are marked as (+). (**C**,**D**) Protective effects and statistics of NAC pretreatment on γH2AX status in MEUB-treated oral cancer cells. Oral cancer cells were pre-treated with NAC (2 mM, 1 h) and post-treated with MEUB (48 h, 0 to 6 μg/mL). Data represent means ± SD (*n* = 3). Treatments labeled with different lower-case letters (a–c) indicate significant differences in a multi-comparison. *p* < 0.05–0.0001.

The DNA damage mechanism for cell killing effects of MEUB was further investigated by γH2AX flow cytometry [30]. In Figure 9A, γH2AX patterns in oral cancer cells after MEUB treatment are shown. In Figure 9B, different treatment times of MEUB induce higher γH2AX (+) (%) than control in oral cancer Ca9-22 and OECM-1 cells.

In Figure 9C, the γH2AX patterns in oral cancer cells are shown after NAC pre-treatment and/or MEUB post-treatment. Figure 9D shows that different concentrations of MEUB induce higher γH2AX (+) (%) than control in oral cancer Ca9-22 and OECM-1 cells. Moreover, this MEUB-induced γH2AX (+) (%) is suppressed by NAC pretreatment.

### 3.11. MEUB Shows 8-OxodG DNA Damage Induction in Oral Cancer Cells

**Figure 10:**
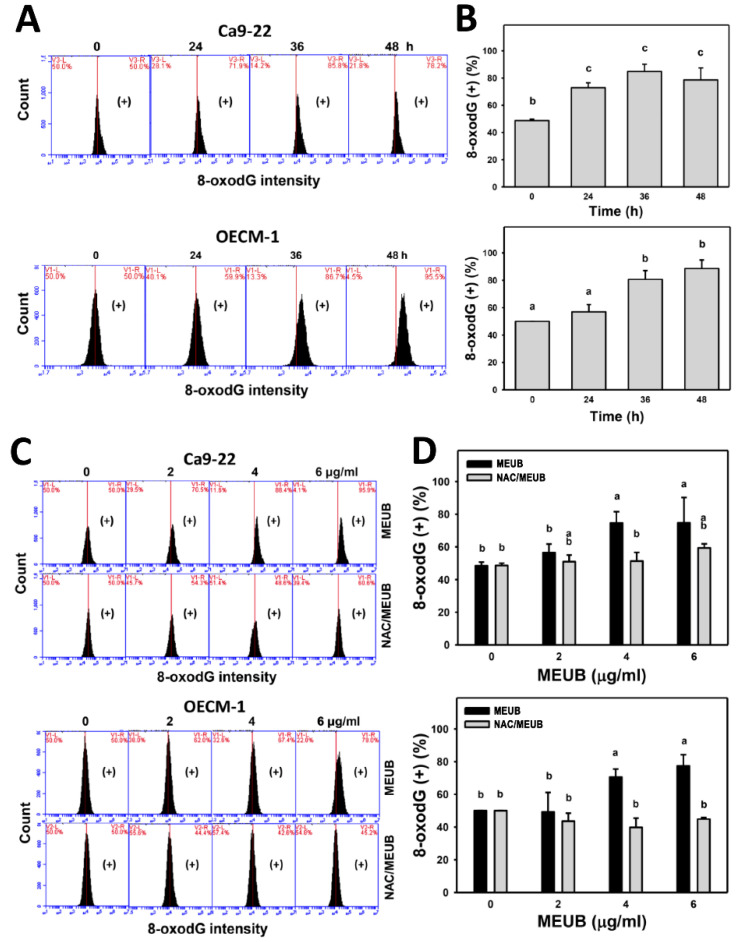
Oxidative DNA damage status of oral cancer cells after MEUB treatments. (**A**,**B**) 8-oxodG patterns of MEUB treatments. Oral cancer cells (Ca9-22 and OECM-1) were treated with MEUB (0 to 48 h, 2 μg/mL). MEUB (0 μg/mL) is the negative control containing 0.1% DMSO in the medium. Cells with high 8-oxodG intensity are marked as (+). (**C**,**D**) Protective effects and statistics of NAC pretreatment on 8-oxodG status in MEUB-treated oral cancer cells. Oral cancer cells were pre-treated with NAC (2 mM, 1 h) and post-treated with MEUB (48 h, 0 to 6 μg/mL). Data, mean ± SD (*n* = 3). Treatments labeled with different lower-case letters (a–c) indicate significant differences in a multi-comparison; *p* < 0.05–0.0001.

The DNA damage mechanism for cell killing effects of MEUB was further investigated by 8-oxodG flow cytometry [31]. In Figure 10A, 8-oxodG patterns in oral cancer cells after MEUB treatment are shown. In Figure 10B, different treatment times of MEUB induce higher 8-oxodG (+) (%) than control in oral cancer Ca9-22 and OECM-1 cells.

In Figure 10C, the 8-oxodG patterns in oral cancer cells are shown after NAC pre-treatment and/or MEUB post-treatment. As shown in Figure 10D, different concentrations of MEUB induce higher 8-oxodG (+) (%) than control in oral cancer Ca9-22 and OECM-1 cells. Moreover, this MEUB-induced 8-oxodG (+) (%) is suppressed by NAC pretreatment.

## 4. Discussion

Lichens contain diverse species and exhibit multiple functions such as in traditional medicine, and the preparation of food, perfume, and dye [32]. Recently, the cell killing effect of lichen extracts has received attention in the treatment of several types of cancer as shown by several studies [11,12,13] except for oral cancer. In the current study, we investigated the anticancer effect and mechanism of MEUB against oral cancer cells. The role of preferential killing, oxidative stress, apoptosis, and DNA damage in oral cancer after MEUB treatment are discussed in the following.

### 4.1. Comparison of Drug Sensitivity between Different Usnea Species Extracts to Cancer Cells

Several extract types of different *Usnea* species showed cell killing effects against several types of cancer cells. For example, methanol extract of *U. intermedia* was cytotoxic against MCF7, MDA-MB-231, A549, and H1299 cells (IC_50_ values were 17.5, 3.0, 21.4, and 10.2 μg/mL in a 72 h ATP assay, respectively) [11]. Methanol extract of *U. filipendula* Stirt induced cell killing against lung (A549, PC3), liver (Hep3B) and rat glioma (C6) cancer cells [13], i.e., IC_50_ values were 37.0, 32.9, 60.5, and 67.9 μg/mL in a 72 h ATP assay. Using acid phosphatase assay at 24 h, different extracts of *U. barbata*, including (E1) CO_2_ supercritical extract and (E2) ether fraction of Soxhlet extract, demonstrated cell killing of mouse melanoma B16 and rat glioma C6 cells, i.e., their IC_50_ values were (E1) 31.21 vs. 58.20 and (E2) 43.40 vs. 69.10 μg/mL, respectively [14].

In the current study, IC_50_ values for MEUB-treated oral cancer OECM-1, Ca9-22, CAL 27, and HSC3 cells at 48 h MTS assay were 1.4, 2.6, 3, and 6 μg/mL, respectively. Therefore, MEUB exhibits higher drug sensitivity to human oral cancer cells than extracts from other *Usnea* species to non-oral cancer cells [11,13,14]. For comparison with other clinical drugs, the IC_50_ value of cisplatin in oral cancer OECM-1 and Ca9-22 cells are 2.61 and 1.20 μg/mL in a 48 h MTS assay (Figure 2B). At 48 h MTS assay, the IC_50_ value of cisplatin in breast cancer SKBR3 and MCF7 cells are 3.71 and 10.45 μg/mL, respectively [33]. Accordingly, the crude extract MEUB shows a similar sensitivity to cisplatin in oral cancer cells and a higher sensitivity than in breast cancer cells.

### 4.2. MEUB Exhibits a Preferential Killing Effect to Oral Cancer Cells

For the drug safety concern, we found that MEUB kept over 95% viability in normal oral HGF-1 cells, suggesting that MEUB is nontoxic to normal cells. Therefore, MEUB exhibits preferential killing to oral cancer cells which provides low levels of cytotoxicity to normal oral cells. The preferential killing ability of extracts from other *Usnea* species are also reported in non-oral cancer cells. For example, water extract of *U. longissima* Ach. exhibits growth inhibition of human glioblastoma GBM U87MG cells but it shows less damage to primary mixed glial-neuronal (PMGN) non-cancer cells [12], suggesting a preferential killing effect on human glioblastoma cells. The preferential killing ability of extracts from other *Usnea* species were also reported from animal model study. For example, ethyl acetate extract of *U. longissima* exhibits antitumor effect against *N*-methyl-*N*’-nitro-*N*-nitrosoguanidin (MNNG) induced on esophagogastric tumor in rats and it is nontoxic to high doses up to 2000 mg/kg [34].

### 4.3. MEUB Triggers Oxidative Stress on Oral Cancer Cells

Some studies using extracts from lichen species exhibit antioxidant properties and cell killing effects to non-oral cancer cells [14,35]. However, the authors focused on several antioxidant abilities but only showed an MTT assay for cell killing evidence to cancer cells. In addition to antioxidant evaluation, the oxidative stress induced by *U. barbata* was reported in the CO_2_ supercritical extract but only ROS change were examined [14]. The antioxidant exhibited a concentration effect to decrease or increase oxidative stress in cells [10]. Moreover, the drug-induced exogenous oxidative stress may induce preferential killing to cancer cells but show low toxicity to normal cells [36]. Therefore, the antioxidant in several *Usnea* species may play a role in preferential killing to several types of cancer cells [12,34].

Although ROS generation has been reported in CO_2_ supercritical extract of *U. barbata* [14], we further reported that different concentrations and treatment times of MEUB can induce cellular ROS and MitoSOX generation as well as MMP depletion in the example of two oral cancer cell lines (OECM-1 and Ca9-22). These results suggest that oxidative stress is highly associated with the cell killing effect of MEUB in oral cancer cells.

### 4.4. MEUB Triggers Apoptosis and DNA Damage on Oral Cancer Cells

Several extract types of different *Usnea* species reported apoptosis and DNA damage on non-oral cancer cells. For example, methanol extracts of *U. intermedia* triggered apoptosis in human breast and lung cancer cells [11]. Methanol extracts of *U. filipendula* Stirt induced apoptosis-like cell death and comet assay-detected DNA damage in human lung, liver (Hep3B), and rat glioma (C6) cancer cells [13]. Similarly, MEUB induced apoptosis in a dose- and time-dependent manner as evidenced by the increase of the subG1 population, annexin V expression, and pancaspase activation.

For DNA damage, MEUB induced DNA damage in the examples of DNA double strand breaks and oxidative DNA damage based on flow cytometry for γH2AX and 8-oxodG. Since 8-oxodG is the marker for oxidative DNA adducts [37], the MEUB-induced oxidative stress may partly contribute to DNA damage.

### 4.5. NAC Recovers MEUB-Induced Apoptosis, Oxidative Stress, and DNA Damage on Oral Cancer Cells

All MEUB triggering changes such as cell killing, subG1 accumulation, apoptosis (annexin V detection, pancaspase activation, and PARP cleavage), oxidative stress (ROS, MitoSOX, and MMP), and DNA damage (γH2AX and 8-oxodG) in oral cancer cells were suppressed by NAC pretreatment. These results suggest that MEUB induces an oxidative stress-mediated mechanism to induce cell killing, apoptosis, and DNA damage in oral cancer cells.

### 4.6. Compounds of Usnea Lichens with Anticancer Effect

In the example of *U. aciculifera*, a number of compounds were identified and some of them, such as aciculiferin A, barbatinic acid, and diffractaic acid, showed a cytotoxic effect to breast cancer MCF7 and cervical HeLa cells, i.e., IC_50_ values at 48 h SRB assays are about 76, 51, and 90 μg/mL, respectively [38]. Although usnic acid is one of the main bioactive compounds in *U. barbata*, it respectively shows high IC_50_ values to mouse melanoma (B16) and rat glioma (C6) cells, i.e., 49.14 and 56.83 μg/mL at 24 h acid phosphatase assay [14]. IC_50_ values of usnic acid for human colorectal HT29, gastric AGS, lung A549, and prostate CWR22Rv-1 cancer cells are 32.8, 5.2, 22.4, and 8.4 μg/mL, respectively [39], while the IC_50_ values for CO_2_ supercritical extract and ether fraction of Soxhlet extract of *U. barbata* are 31.21 vs. 58.20 μg/mL, respectively. These findings suggest that crude extracts may exhibit a higher drug effectivity than their individual bioactive compounds. It is possible that crude extracts provide multiple targeting effects by different compounds and generate a synergy which is a valuable condition in cancer therapy.

## 5. Conclusions

The antioral cancer effect of extracts from *Usnea* lichens was rarely investigated. In the present study, we evaluated the cell killing effect of MEUB and explored its cytotoxic mechanism in oral cancer cells. MEUB demonstrates the potential to preferentially kill a number of oral cancer cell lines without reducing cell viability of a normal oral cell line. MEUB overproduces oxidative stress including ROS and MitoSOX productions as well as MMP depletion. Moreover, MEUB exerts apoptosis, DNA double strand break damage, and oxidative DNA damage in oral cancer cells. All these changes for cell killing, oxidative stress, apoptosis, and DNA damage induced by MEUB were recovered by antioxidant NAC pretreatment. We conclude, therefore, that MEUB induces cell killing, apoptosis and DNA damage against oral cancer cells through oxidative stress.
